# Using group model building to frame the commercial determinants of dietary behaviour in adolescence – proposed methods for online system mapping workshops

**DOI:** 10.1186/s12874-022-01576-y

**Published:** 2022-03-27

**Authors:** Yanaina Chavez-Ugalde, Zoi Toumpakari, Martin White, Frank De Vocht, Russell Jago

**Affiliations:** 1 NIHR School for Public Health Research, Bristol, UK; 2 Bristol Medical School, Population Health Sciences, University of Bristol, BG3, Oakfield House, Bristol, BS8 2BN UK; 3 Centre for Exercise, School for Policy Studies, Nutrition and Health Sciences, University of Bristol, Bristol, UK; 4 MRC Epidemiology Unit and Centre for Diet & Activity Research, University of Cambridge, Cambridge, UK; 5 NIHR Applied Research Collaboration West (NIHR ARC West), Bristol, UK

**Keywords:** Group Model Building, Online workshops, Adolescence, System mapping, Public health, Dietary behaviour, Causal loop diagram

## Abstract

**Background:**

Group model building (GMB) is a participatory approach whereby diverse stakeholders can share their views about a problem to create a collective understanding of a complex system. In this article we report our methodological approach to adapt face-to-face GMB processes to an online format to explore the mechanisms by which commercial drivers influence adolescents’ dietary behaviour. We use our experiences to make recommendations on how online GMB could be delivered.

**Methods:**

We planned, adapted, piloted, delivered, and evaluated a series of online GMB workshops with adolescents, policymakers and public health practitioners to create a system map of the commercial determinants of dietary behaviour in adolescence. We adapted face-to-face GMB workshops to a series of 3 online GMB workshops with 11 adolescents (16–18 years) living in the Southwest of England, and one GMB workshop with policymakers and public health practitioners.

**Results:**

In our experience, adapting, and delivering GMB online is feasible, engaging, cost-saving and an enjoyable experience. Participants gave positive feedback in terms of engagement and enjoyment, and it allowed them to recognise different points of view about the same problem. Participants became familiarised with system thinking and system dynamics concepts, developed a shared understanding of a complex issue and portrayed it in a system map that depicted the most important factors in a causal structure as well as their interactions at various levels.

**Conclusions:**

We recommend using process mapping to understand the overall GMB process in an online environment and piloting the workshops to test the timings and flow between online platforms. For facilitation and delivery, facilitators need to ensure they can create an inviting and engaging online environment, even for participants who decide to have their cameras off. Separating GMB activities into different workshops allowed participants to reflect on the problem being discussed and bring new ideas to subsequent workshops. Evaluating the workshops enabled us to build evidence on the trade-offs between the effectiveness, quality and efficiency of online GMB workshops, and how this might be enhanced to identify leverage points and achieve systemic changes in complex issues.

**Ethical approval:**

The research was approved by the University of Bristol’s Faculty of Health Sciences Research Ethics Committee (Ref: 99,003) and written consent was received from all participants.

## Background

Complex systems thinking has gained popularity among public health researchers [1, 2]. This is partly because traditional evidence models have been criticised for being ill-suited to account for the “real-life” contexts in which health decisions are made and the broader systems in which policies and interventions take place [3, 4]. Therefore, a “complex systems approach” has been advocated as a way of understanding the many influential factors and different sectors, and their interactions that affect complex health issues like diet and obesity [5]. Incorporating systems thinking methods into policymaking has the potential to increase policy efficacy and better anticipate unintended consequences of well-intentioned practices from other contexts [6]. However, the applications of systems science methodologies in public health research are relatively underdeveloped [7]. 

The commercial determinants of obesity are strategies used by the food and beverage industry to produce, promote and increase the sales of their products, sometimes at the expense of public health, especially where their promotion strategies particularly target children and adolescents with foods high in salt, fats, sugar and energy density through integrated marketing techniques [5]. Incorporating systems thinking can actively foster dialogue between diverse stakeholders and enlighten the mechanisms by which commercial drivers influence core dietary behaviour and can affect health a long time into the future.

Group Model Building (GMB) is a facilitated modelling method based on system dynamics (SD) [8] whereby system actors (i.e. stakeholders from civil society, academia, policymaking and business) share their views and ideas about a problem to create a collective understanding of a complex system [9]. The aim of GMB is to uncover the causal structure of complex systems driving its outcomes [8]. The causal structure of the system is presented in the form of a Causal Loop Diagram (CLD), or system map, which provides a visual representation of the most influential factors, their interconnections, and feedback structures in the system that are thought to be responsible for creating a problem, or making an existing problem worse. It also specifically identifies what factors are included and excluded in the system of interest (i.e. its boundaries), and enables the identification of leverage points at which system change can potentially be initiated.

Existing literature on conducting GMB workshops mainly focus in in-person facilitation [10, 11], which has been successful in fostering discussion among stakeholders from multiple sectors and with different beliefs and values on a topic [12]. However, with technological advancements in recent years, and the impact of COVID-19 pandemic, other methods for developing CLDs of systems are gaining attention, including digital collaborative tools, online discussion environments and meeting platforms [13]. Conducting GMB workshops online may have several advantages, such as reduced costs and time required to run the workshops, inclusion of a broader range of stakeholders regardless of their physical location and breaking down GMB activities into several short online sessions rather than whole day workshops, which would allow for reflective processes between sessions. Despite the aforementioned advantages, we only found two examples of fully online GMB workshops in the literature [14, 15], which were held online due to COVID-19 restrictions.

We ran a series of Group Model Building (GMB) workshops to create a system map of the commercial determinants of dietary behaviour associated with obesity and, due to the COVID-19 global pandemic, we assessed adapting and running the GMB workshops fully online. Specifically, we ran GMB workshops with adolescents (16–18 years) living in the Southwest of England and aimed to create a system map of the of the commercial determinants of dietary behaviour associated with obesity, and to encourage them to identify and formulate relevant policy ideas to tackle these commercial influences and to improve their food choice environment. In a second workshop, we gathered policymakers and public health practitioners to discuss the system map created by the adolescents and explore the potential for the implementation of some of the policy ideas proposed at a local authority level.

The detailed account of the results from the workshops will be presented elsewhere, but for illustrative purposes, we will present the final map here. The aim of this paper is to report on our experiences of adapting face-to-face GMB processes to an online format, with a particular focus on our methodological approach. We use our experiences to produce recommendations on how online GMB can be delivered.

## Methods

**Fig. 1:**
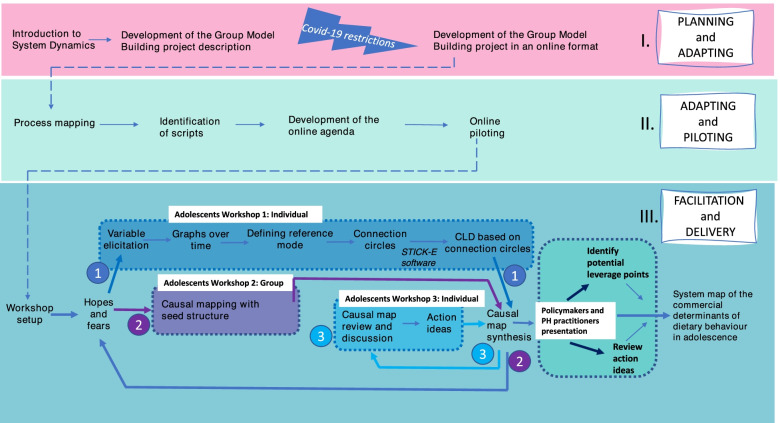
Flow diagram of the GMB process

Figure 1 shows the process we followed to plan, adapt, pilot, and deliver the GMB workshops with adolescents, policymakers and public health practitioners. In a first phase (Fig. 1*. I. Planning and Adapting)*, because of COVID-19 restrictions on running face-to-face activities, we had to develop an adapted version of the GMB project for an online format. In a second phase (*II*. *Adapting and Piloting*), we conducted a process mapping exercise to develop a shared understanding of the overall GMB process, discuss the number of workshops, and select which and how many people would be involved in each one. The activities were based on standard scripts that outline the elements to include in a GMB workshop [16, 17]. We identified the scripts to include in the GMB workshop and then developed the online agenda and the timings for each activity. To avoid “screen fatigue” we divided the GMB workshop with adolescents into 3 shorter online workshops, i) the introductory workshop, ii) the GMB workshop with all the adolescents and iii) a validation workshop. We adapted the scripts to a single one-hour online workshop with the policymakers and public health practitioners since the time available from this group was limited due to COVID-19 response requirements. To test the online platform and the timings for the activities, we did two online piloting workshops with volunteers from the University of Bristol. In a third phase (*III. Facilitation and Delivery*) we delivered the three GMB workshops with adolescents (1. Introduction – individual; 2. GMB – group; and 3. Validation – individual), and the one workshop with policymakers and public health practitioners.

Since we had two very distinct groups of stakeholders, we had a few shared objectives and some specific **objectives** for each group:

**Shared objectives:**

- Familiarise participants with system thinking and system dynamics concepts (e.g. interconnections, feedback loops, time delays).
- Develop a shared understanding of a complex issue (i.e. the commercial determinants of dietary behaviour associated with obesity in adolescence) and portray it in a system map that depicts the most important factors and how they interact at various levels.
- Encourage participants to see the “big picture” and think about ways to intervene at the system level to achieve change.

**Specific objectives for adolescents:**

- Build a system map that depicts their views of the most important factors, shaped by the food and beverage industry, that influence what they choose to buy and eat.
- Encourage them to think about policy / solution ideas to the problem to achieve a systemic change.

**Specific objectives for policymakers and public health practitioners:**

- Share the system map and policy ideas created by adolescents and compare their views on how food and beverage industries influence what adolescents choose to buy and eat.
- Discuss the most explored / and unexplored areas on the map in policymaking efforts.
- Discuss barriers to implementation of policies that could have the highest impact on adolescent’s dietary behaviour and obesity.
- Explore the possibility of using the system map as a communication tool to incorporate the commercial determinants of dietary behaviour into current public health thinking.
- Explore the potential for the implementation of some of the policy ideas proposed at a local authority level.

The GMB workshops were based on Scriptapedia [16, 18], an online freely distributed book which uses scripts for structured group exercises as guides to conduct GMB practice. The process mapping enabled drafting the different activities required to be able to build the system map with the adolescents. We used *BlueJeans*, a virtual online platform, and *STICK-E *[19], a licenced application developed by system mapping experts in Deakin University, Australia. Table 1 gives a brief explanation of the Scriptapedia scripts we used.

**Table 1:** Scriptapedia scripts used and aim of each script

Scriptapedia script name	Aim of script
Process mapping	This script is used at the beginning of the GMB planning. It helps in planning and developing a shared understanding of the GMB process, select the number of workshops needed, and identify the number of participants you would like to involve in each workshop and establish the inputs and outputs for each workshop
Hopes and fears	This script is used at the beginning of the GMB workshop to determine group expectations
Variable elicitation	This script is used at the start of the system building phase. It prompts group discussion about the problem, the elements the group believes causes or contributes to the problem, and helps to set the boundaries of the system
Graphs Over Time	This script is used at the beginning of the GMB workshop as it aims to engage participants in a more detailed discussion about the problem. It is used to frame the problem, elicit potential influential variables, and decide the reference modes for the workshop
Connection Circle	This script it used to visualise the variables and the interconnections between the variables participants believe to be important in causing or contributing to the problem
CLD from Connection Circle	This script is used after visualising the variables in a connection circle. It helps to create a CLD by identifying the hypothesized causal relationships between variables and feedback loops
Causal Mapping with Seed Structure	When there is an initial causal structure of the problem being discussed (from a review of the literature or previous workshops), this script helps to exemplify how the problem involves a system of interacting feedback loops
Model Review	This script is used at the end of the workshop to recapitulate the story behind the variables and their connections, it helps to explain anything that was left unclear, include any additional information, and it prompts feedback from participants
Action Ideas	This script is used after the system map (model) has been reviewed and developed to identify and prioritise potential actions (or policies) to intervene in the system

### Participant recruitment

Adolescents had to be between 16–18 years of age, live in the Southwest of England, have access to a stable Wi-Fi connection and be willing to participate in all 3 online workshops to be included. We reached out to 13 youth groups based in the Southwest of England. Only 3 responded [Bristol Young People’s Advisory Group (YPAG), Avon Scouts, and Knowle West Media Centre] and we recruited 11 adolescents, 10 from YPAG and 1 from Avon Scouts. Participants were offered a £30 online Amazon voucher for participating in the 3 online workshops.

Public health practitioners and policymakers were recruited through existing network of contacts (i.e. University of Bristol, NIHR Applied Research Collaboration (ARC) West). Due to COVID-19, the time available from this group was limited, therefore we only had a one 1-h workshop.

The research was approved by the University of Bristol’s Faculty of Health Sciences Research Ethics Committee (Ref: 99,003) and written consent was received from all participants.

### Adaptation of face-to-face GMB workshops to an online format

**Fig. 2:**
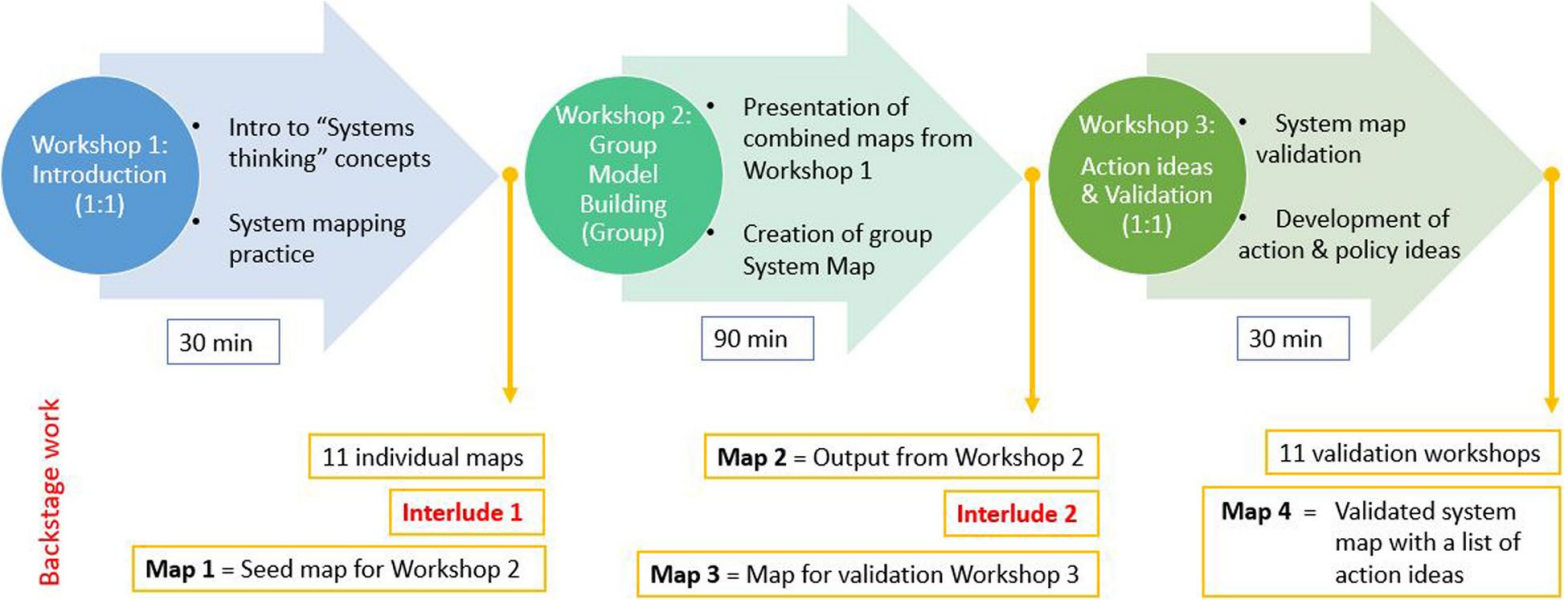
Adaptation of adolescents’ GMB workshops to an online format

Participants were invited via email with instructions to fill out a brief demographic questionnaire and a consent form to participate in the GMB workshops. The three online workshops with the adolescents had the following structure (Fig. 2). The first workshop aimed to introduce participants to system thinking concepts and build a system map; during the second workshop the adolescents, as a group built the system map; in the third workshop participants validated the system map and confirmed that the factors they mentioned in workshop 2 were portrayed in the map in the way they meant them to be and were encouraged to share action or policy ideas to intervene in the system. Table 2 shows a detailed summary of the three online GMB workshop activities.

**Table 2:** Summary of the three online GMB workshop activities

**Workshop 1**: Introduction—Individual meeting with participant (30 min) Participants: Adolescent and modeler/facilitator		
**Activity script** (time)	**Aim and description** (applications used)	**Output**
Introduction to systems and defining terms (10 min)	Presentation of workshop agenda and objectives Introduction to systems thinking, defining system dynamic concepts (variables, connections, polarity, feedback loops) (BlueJeans, PowerPoint)	Individual CLDs representing, at high level, which activities and how commercial food and beverage industries influence adolescents’ dietary behaviour
Hopes and fears (2 min)	To prompt and establish participant expectations for the workshop (BlueJeans, PowerPoint)	
Variable elicitation (5 min)	Participants named factors that responded to the prompt: “**WHAT** do you think food and beverage companies (companies, shops, online platforms) do to have an impact on what you / your friends choose to buy and eat.” The factors mentioned were annotated into STICK-E, a system mapping platform (BlueJeans, PowerPoint, STICK-E)	
Behaviour over-time graphs (3 min)	Participants were prompted to identify key factors around the problem and their development over time. Participants were prompted to draw the behaviour over-time graphs in a digital white board. This activity also helped in gathering insight in deciding the reference modes for the mapping exercise (BlueJeans digital white board, PowerPoint)	
Connection circle (5 min)	The shared screen moved from PowerPoint to STICK-E. The factors mentioned during the variable elicitation exercise were annotated into STICK-E and participants were guided to identify the connections between the different factors that contribute to or are affected by the problem variable (BlueJeans, STICK-E)	
Causal Loop Diagram from connection circles (5 min)	Using STICK-E, the connection circle can be transformed into a CLD and participants were guided to describe the causal structure and feedback relationships between the variables. Participants were prompted to think about the nature of the relationship between the variables and to visualize various interactive causal pathways affecting their dietary behaviour (BlueJeans, STICK-E)	
**Interlude 1**: CLD synthesis—Modeller/facilitator		
**Activity**	**Aim and description** (applications used)	**Output**
Causal map synthesis	Combine the CLDs produced in Workshop 1 into an overarching CLD containing mentioned factors/variables and connections (STICK-E)	Synthesised CLD with seed structure for Workshop 2 -Map 1
**Workshop 2: Group Model Building workshop** – Group participants (90 min) Participants: Adolescents, modeller/facilitator and note taker		
**Activity script**	**Aim and description** (applications used)	**Output**
Causal mapping with seed structure	To develop further the CLD created in Workshop 1	A raw overarching CLD that reflects the group views of the problem and represent it in a system of interacting feedback loops -Map 2
(10 min)	Modeller/facilitator explained the aims of the workshop and had a refresher of concepts	
(15 min)	Participants were asked to review the synthesised CLD, prioritise variables and identified overarching themes	
(15 min)	Participants were asked to share additional variables that came to mind between Workshop 1 and 2, explain causal connections between the variables. Recorder documented working definitions, variable connections, and key words	
(30 min)	Participants were prompted to examine the structure of the CLD, encouraged to change, add, or correct any misrepresentations of variables and loops in the model	
(20 min)	Participants identified and narrated the feedback loops in the CLD. As participants reviewed the model, the facilitator/modeller revised the causal structure and shared in-real time the latest version of the CLD (BlueJeans, PowerPoint, STICK-E)	
**Interlude 2**: CLD synthesis—Modeller/facilitator		
**Activity**	**Aim and description** (applications used)	**Output**
Analysis of recordings and narratives. Causal map refinement	The recording and the map created in Workshop 2 were analysed to make sure that the variables and connections narrated and mentioned in the workshop were reflected on the map (Workshop recording, STICK-E)	An overarching CLD created by the group -Map 3
**Workshop 3:** Causal map review, validation, and action ideas – Individual meeting with participant (30 min) Participants: Adolescent and modeller/facilitator		
**Activity script**	**Aim and description** (applications used)	**Output**
Model review (15 min)	The modeller/facilitator showed the overarching CLD created by the group. Participants were prompted to validate if the refined map from Workshop 2 represented their ideas and how they meant for them to be represented Key feedback loops were articulated, and narratives and themes were clarified Modeller/facilitator confirmed participants’ understanding of the correspondence between model structure and system behaviour (BlueJeans, PowerPoint, STICK-E)	A CLD revised and validated by workshop participants -Map 4
Action ideas (15 min)	Participants were prompted to generate a list of intervention / policy ideas targeting the causal structure of the CLD (variables, connections, rules that govern the connections, goals in the system, mindset.) Each action included: -A description of the action -The place where it would impact the system map -Identify how hard or easy it would be to implement (easy – hard) -If successfully implemented, the level of impact it might have on the system (low – high impact) (BlueJeans, PowerPoint, STICK-E)	A list of intervention options targeting variables, connections or rules that govern the connections, goals in the system or mindset A description of how they might affect the system
**Post- workshop**: Evaluation forms		
**Activity**	**Aim and description** (applications used)	**Output**
Evaluation (5 min)	This was a brief questionnaire to evaluate strengths, weaknesses of the workshops and to assess participants’ enjoyment, engagement, satisfaction, and learning outcomes (Microsoft Forms)	An assessment of the workshops’ strengths, weaknesses, and participants’ experience in taking part in the GMB workshops

### Evaluation and validation

**Fig. 3:**
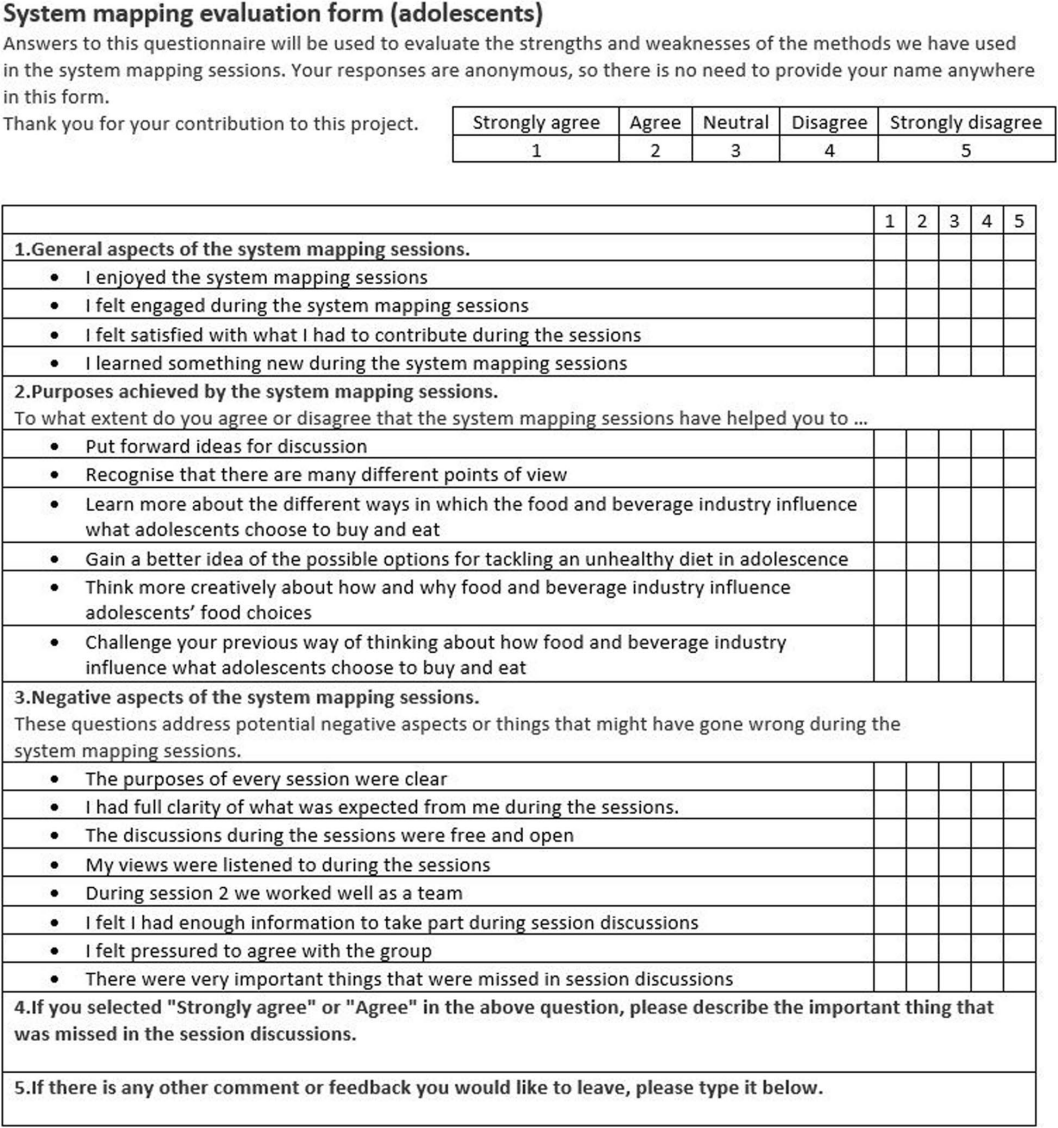
Evaluation form

Participants were asked to fill out a brief anonymous online feedback survey to evaluate strengths and limitations of the workshops and to assess participants’ experiences in taking part (see Fig. 3). The survey had five sections, and the first three were answered through a 5-point Likert-scale ranging from “strongly agree” to “strongly disagree”. The first three sections evaluated general aspects of the workshop, if they believed that the aims were achieved, and if they thought there were any negative aspects of the system mapping workshops. The last two sections allowed participants to report any important things that were left out from the discussions and to add any further comments or feedback about the workshop.

As suggested by previous research on methodological evaluation of participatory system mapping methods [20], our evaluation included questions to evaluate whether the method was well suited and engaging for all participants, regardless of background or previous knowledge (Sect. 1 of the survey), and whether the process itself was suitable for participants to have the freedom to introduce new topics (Sect. 2 and 3). A third important aspect of system mapping evaluation is to explore if the GMB workshop had the potential to influence decision and policy making. This was explored verbally with the policymakers during the session.

We used two approaches to assess validity of the system map. First, the most influential factors were developed in an inductive manner during the modelling sessions with the adolescents. These were then validated through internal consensus and checked against scientific literature to support external validity [21]. Internal consensus was assessed verbally (with the adolescents as a group in workshop 2, and then with each adolescent individually in workshop 3; and during the policymakers’ session) by asking participants if they agreed that the map portrayed the most important influential factors, interconnections and feedback loops in how food industry has an influence over what adolescents choose to buy and eat. In case of non-agreement, the map was modified accordingly in real time until agreement was reached.

## Results

### Facilitation and delivery of online GMB workshops

- Adolescents

**Fig. 4:**
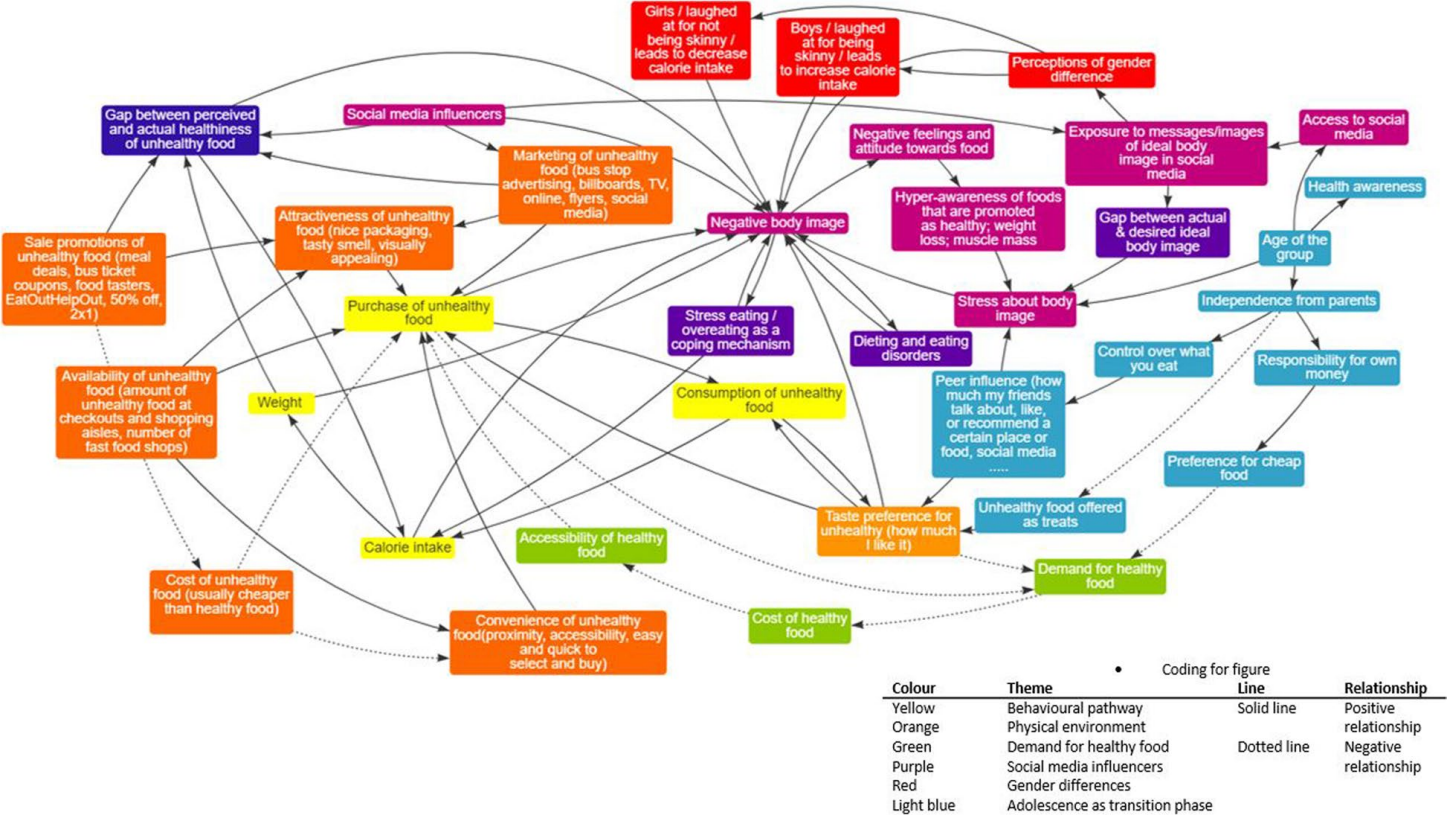
GMB validated system map created by the adolescents

During the three workshops the facilitator made sure everyone’s ideas were heard and included in the discussions on what to include in the system map. They shared different perspectives about how the food and beverage industry influence their food choices, and the different ideas they thought could tackle unhealthy diets in adolescence. Adolescents became familiarised with system thinking and system dynamics concepts, developed a shared understanding of a complex issue and portrayed it in a system map that depicted the most important factors in a causal structure as well as their interactions at various levels (see Fig. 4). The system map created by adolescents had 70 causal links which connected 37 elements and had 7 feedback loops. The 37 elements were grouped into 6 themes or pathways, represented by the different colours in the map. Adolescents understood feedback loops as the circular nature of cause and effect, and they exemplified loops as the “chicken or egg” metaphor. Based on the map, they were also familiar with reinforcing loops—an action that creates a result which produces more of the same action, resulting in continued growth or decline; and balancing feedback loops – an action that creates a result which produces the opposite direction of the initial action, redirecting the system towards equilibrium [22].

- Policymakers and public health practitioners

This was a 1-h workshop where we discussed the validated system map created by adolescents (Map 4) and shared the policy/action ideas they suggested. During the workshop participants added factors to the map based on their expert knowledge and their views and identified areas of current policy intervention as well as unexplored areas with the aim to identify potential leverage points. They did not remove or changed any of the arrows or factors in the map. Participants were encouraged to suggest any additional factors they thought were important and not included in the system map made by the adolescents. These were included in real time using STICK-E. Participants validated verbally that the system map created by the adolescents was in agreement with their mental models. The key feedback loops in the map were articulated and the modeller/facilitator confirmed participants’ understanding of the correspondence between model structure and system behaviour. This led to the final overarching system map of the commercial determinants of dietary behaviour in adolescence. During the workshop we explored the possibility for participants to use the system map as a communication tool to incorporate the commercial determinants of dietary behaviour into current public health thinking, and identify whether it had the potential to influence policy making efforts.

### Practicalities of adapting GMB workshops to an online format

- Technical issues

Online delivery of a workshop requires a platform that is both safe (with respect to confidential information and ensuring non-invited people are not able to log on) and sufficiently flexible to allow for interactive activities (e.g. drawing graphs over-time, mapping the system in real time) in an engaging layout [23]. At the time of the workshops the University of Bristol considered BlueJeans to be the most secure platform. We also needed an engaging platform where we could build and share the system map in real time. STICK-E is a platform that was designed to facilitate community knowledge exchange and promote a shared understanding of complex problems and fulfilled our needs for the workshops.

- Costs

We initially budgeted costs for room hiring (£150 per workshop × 2), refreshments (£150 per workshop × 2), stationary materials (£50), and facilitator and note-taker travel costs (£7 per day × 2). We did not include travel costs for participants since the workshops were going to be held near or at their school or council offices. Delivering the workshops online allowed us to save approximately £664 since we did not have to hire any rooms for the workshops, provide refreshments, spend on travelling costs, or purchase any stationary material.

- Participants

We developed the online agenda aiming to minimise participants’ burden and cater for dates and times that would work for each one of them. For example, some adolescents were still in term time when the workshops happened, and we had to offer out-of-office hours and weekends so they could choose a date and time that was convenient for them. Similarly, policymakers had very limited time available due to the duties they had in responding to the COVID-19 pandemic and we had to design it to fit into a one-hour long workshop. Piloting the workshops was an essential part of the planning and adapting phase. Even though the GMB scripts were a good guide to design the online workshops, we had to test, from a participant’s perspective, that the workspace, the transition between platforms and the different workshops activities were engaging and closely connected. We piloted the workshops with postgraduate students from the University of Bristol and Cambridge. This allowed us to readjust the timings of some activities, for example, extend by 15 min the time allocated to do the system map in workshop 2. We were able to recruit 11 adolescents and 6 policymakers and practitioners.

- Workshop management

Running GMB workshops requires multiple roles (e.g. facilitator, modeller, recorder or note-taker) to balance the group process and generate the best possible results [24]. We had two roles during the workshops: modeller/facilitator and note-taker.

The modeller/facilitator was responsible for hosting and moderating the discussions and to guide the group to build the system map. She was trained in GMB methods, had substantive knowledge in the problem being mapped, and had strong group facilitation skills. She participated in all 3 workshops with the adolescents and the workshop with the policymakers. Participants can have different levels of engagement with technology and online platforms and the modeller/facilitator needed to find the balance between letting participants share and expand on their views and keeping within the boundaries of the problem being addressed. Time pressure, participants’ screen fatigue and engagement were important factors to account for in an online context since it limits the facilitator’s capacity to read nonverbal cues which are easier to manage in face-to-face interactions. To compensate for this the facilitator had to check-in regularly and having an initial individual workshop with each of the adolescents helped in building a good rapport with them before the group activity and enhanced an active participation throughout workshop 2 and 3. Having an additional facilitator would have been useful to keep detailed observation of participants’ engagement and interaction. Some participants were more comfortable in speaking up, while others were more reticent. To allow for all participant to speak up the facilitator was proactively asking specific participants to share their thoughts. Online workshops tend to demand more energy from participants than face-to-face workshops, and it was an important factor when designing the workshop agenda.

The note-taker only participated in workshop 2 and was responsible for recording key interactions between participants, as well as non-verbal expressions and key phrases. This added context to the recordings and allowed us to underline key points mentioned by the participants. She was also able to pause the workshop to clarify any terms or narratives that were not clear. After the workshop the modeller/facilitator and the note-taker worked together to consolidate the notes and make sure that participants’ conversations (verbal and non-verbal expressions) and key points were included in the final system map.

- Evaluation and validation

Almost all the adolescents (10/11), and all the policymakers and practitioners responded to the survey (6/6). Overall, participants enjoyed the workshops, felt engaged and satisfied with what they had to contribute and believed they had learned something new. In terms of achieving the intended aims of the workshops, participants felt that they were able to put forward ideas for discussion, recognised there were many different points of view, were prompted to think more creatively about the problem being addressed and slightly challenged their previous ways of thinking about the problem.

In terms of methodological evaluation of the system mapping workshops, the survey indicated that the method was well suited for young people and practitioners and that they were given freedom to introduce new topics and cognitive frames. The policymakers discussed the potential for the system map to be used as a communication tool to visualise the complexity, but also the potential leverage points for intervention in policy making efforts.

Validation of the map occurred through internal consensus, first as a group, and then individually by adolescents. When adolescents wanted to add elements or change direction of association during the workshops, changes were made in real time. We considered full validation of the map to be when the entire group agreed with the map. We did not encounter any disagreement or non-validation from the adolescents neither during the group nor the individual workshops.

The map validated by adolescents was shown to policymakers and public health practitioners, and they further validated it by verbally confirming the factors and interrelations highlighted by the adolescents. After policymakers and public health practitioners validated the map, we used this as the basis for further discussion of the complexity of the influence of the food and beverage industry on adolescents’ dietary behaviour. Policymakers highlighted the difficulty of simplifying the complexity of the issue into a diagram, but also the methodological value of visualising the factors and interconnections in a map to uncover causal links and use it as a decision-making tool.

Participants provided feedback that key strengths of the workshops were that the purposes of every workshop were clear and they had clarity on what was expected from them, that the discussions during the workshops were free and open and that their views were listened to without feeling pressure to agree with the group, and that nothing important was left unsaid. Below are some quotes from the adolescents’ feedback illustrating this:

*The researcher was very friendly and listened well, giving everyone a chance to speak in group workshops and asking if there was anything you wanted to say in the 1-1 workshop! I am very positive about my experience.*

Adolescent participant 1.

*I think that the meetings were very relaxed and I really enjoyed them and I was made to feel like what I was saying was helpful which was encouraging.*

Adolescent participant 2.

Policymakers and public health practitioners also enjoyed the workshop. However, in terms of timings they felt that they needed more time to discuss the map and the adolescents’ policy ideas:

*You did a brilliant job of running an engaging session! You kept slide content minimal and talked through everything very clearly - clearly a well-planned session. It's definitely hard to get the timing right for a session like this - I think that we could easily have gone in for another hour, but I know that time is always limited. I think for next time, may not need as much time on the introductions, and then you can maximise the time available to discuss your objectives. Top work :)*

Public health practitioner/policymaker 1.

## Discussion

We found many advantages of doing GMB workshops online in terms of cost and commuting time. Initially we costed ≈£650 for room hire, refreshments, stationery and travel costs for the facilitator and note-taker. In addition, the time between each of the workshops with the adolescents allowed for a reflective process to occur making the following workshops more engaging. Adolescents were very keen to share their experiences and interaction with their food environments and how the first workshop allowed them to see their food choice processes more critically, for example, when they went to the shops or used social media, they had a critical eye on the food marketing that “popped-up” on their screens.

We enhanced the development of system’s mapping experience into an online environment [14] by implementing Causal Mapping with Seed Structure and by exploring the use of Graphs Over Time and Action Ideas scripts, as suggested by Wilkerson et al. (2020). We started the GMB workshop’s discussion with a seed structure (i.e. the synthesised map from the 11 individual workshops with the adolescents), which allowed us to quickly illustrate how the problem being discussed involved a system of interacting feedback loops. Using the Graphs Over Time script prompted participants to think in terms of variables (i.e. factors which are amenable to change) and it facilitated the initiation of the mapping process having decided on the variables they thought to be the most important ones. Finally, using the Action Ideas scripts allowed adolescents to think about policy ideas to intervene in the system map they had created. These ideas were then shared with the policymakers and public health practitioners.

Validation of the system map was achieved through consensus among workshop participants and by comparing the map to existing literature on the topic, and the method was evaluated through an anonymous survey at the end of the workshops. These processes could be carried out equally well in an in-person or in an online workshop. However, we do not recommend having a mixed setup (some participants online, and some in person) due to power dynamics and facilitation challenges [14] and because participants online might miss the in-person dynamic, causing an unbalanced communication.

Online GMB should not be thought of a substitution for face-to-face delivery, but rather as a complementary option for system mapping. As more research emerges on this topic it would be valuable to compare and evaluate the effectiveness of online versus in-person GMB workshops in terms of the quality of the results and long-term mental model changes on participants.

We were interested in introducing participants to systems thinking concepts (i.e. interconnectedness, feedback loops), but within the timeframe of the study were not able to also explore concepts of accumulation or feedback loops with time delays, neither could we compare system maps at different time points, between groups or delivery modes (i.e. online vs face-to-face). A deeper understanding of complex systems methodologies, including the aforementioned interacting feedback loops, time-delays, and accumulations, might have further improved the final maps [25, 26].

Compared to face-to-face workshops, the online workshop process could have impacted on the validation by limiting the consensus on relevant variables due to online fatigue, or by making it more difficult for the facilitator to track the non-verbal behaviour of participants. However, previous research suggests that discussions in online workshops, compared to in-person, does not impact on quality while also moderators report less overtalking than in in-person ones [23]. Additionally, we placed particular emphasis on encouraging participants to voice their ideas, both in the group, and subsequently individually in case they did not feel confident in expressing their ideas in the group. Therefore, we believe that our methodological evaluation supports our study’s findings. However, we recognise that validation methods for GMB models, such as meditation analysis, structural-equation-modelling, exploratory factor analysis could help quantitatively test the relationships among the elements and underlying factors, and account for their non-linear relationships in the model and produce more robust models to support policymaking efforts [15].

Designing and delivering online GMB is achievable, however it comes with its challenges due to the many aspects and roles needed to deliver the workshop activities. We have provided some recommendations to overcome these in Table 3 Additionally, guidance on best practices for online power dynamic facilitation would be beneficial to have more balanced relational processes when performing participatory activities, like GMB workshops, where one of the aims is to ensure everyone has an equal opportunity to share their views about the causes of a problem and its potential solutions to enable a systemic change [27]. Having relatively small groups with minimal levels of power differences seemed to be a beneficial factor when facilitation online GMB workshops. Additionally, having separate workshops with participants to introduce them to system dynamic concepts and to the system mapping platform seemed to have favoured active and confident participation during the group workshop.

**Table 3:** Recommendations to run an online GMB workshop

Planning
1.Do a **process mapping** exercise to understand the overall GMB process, discuss the number of workshops, and select which and how many people would be involved in each one. Involve a manageable number of participants so that the online facilitator has capacity to monitor every participant videos simultaneously and engage with everyone, even with those that prefer to have their cameras off
2.**Pilot** the workshop with the modelling team (at least involve two people rather than just one, we suggest having at least one modeller/facilitator and a note-taker) and volunteers to fine-tune the timings of each activity and test the software/platforms (e.g. STICK-E, MS Teams, Zoom, Miro) you will use during the workshop
**Facilitation and delivery**
3.Ensure the **facilitator** can ensure participant’s engagement throughout the workshop(s) and the team is able to adapt to participants’ requirements
4.Guarantee a that the **facilitator** can create an inviting online environment for participants to share their thoughts as well as mediating **conflicts** that may arise between participants during the workshops
5.In an online environment, make sure the facilitator/modeller is able to **supervise** participants’ videos whilst running the activities and able to engage participants that decide to have their cameras off. This role requires experience not only in conducting in-person workshops but also requires familiarity with an online environment and the platform (s) used (e.g. STICK-E, MS Teams, Zoom, Miro)
6.To avoid screen fatigue, **separate** the GMB activities into multiple workshops, we suggest 60–90 min each a.Have an **introductory** workshop with participants to build rapport with the researcher, get them familiarised with the platform/software, introduce them to “system mapping” concepts and the problem you will be discussing during the GMB workshop, and have a practice workshop to build a system map in real-time (i.e. STICK-E) b.Doing workshops online allows to have follow-up sessions because participants do not need to travel. Having more than one online workshop allows to have **time** in-between (i.e. interlude). This enables participants to have some “reflective” time and incorporate the problem you discussed in the first workshop, expose themselves to their environment and think of any new variables or connections, which they can share in the following workshop c.When running the GMB workshop online, you can have a “refresher” to **remind** them of the problem, the system thinking concepts, and encourage them to share any **new variables** they thought about
7.Once participants think there are enough variables and connections between variables during the GMB workshop, ask participants to **examine the structure of the system map** and add, change or correct any misrepresentation of variables or connections in the map. Since they will have time between workshops, this reflective time can allow them to be more critical when reviewing the system map in the following workshop
8.Once the map seems to be “finalised”, **narrate the story** that the map tells (i.e. how variables are interconnected and the direction they influence each other). You will have time to revisit the narrative during the validation workshop and modify if needed
9.Ask participants to **confirm if the map reflects’ their thoughts** of how they think the system behaves. Again, having an opportunity to revisit the map at a follow-up workshop can help participants to be critical when reviewing the system map
10.Have a final “**validation workshop**” where participants’ analyse the system map and agree that the map reflects’ their thoughts of how the system behaves
11.During the introductory workshop and the GMB, remind participants that at the end you will encourage them to think about **policy ideas or interventions** that target the causal structure of the system map (variables, connections, rules that govern the connections, goals in the system, mindset). This will allow them to reflect on the different policy ideas and share at the final workshop once the system map is finalised
**Evaluation**
12.Have an **evaluation** form where participants can share their experience of the workshop, evaluate the appropriateness of the method, and give feedback on how to improve in future workshops
**Validation**
13.Make sure you have validation methods in place (i.e. ensure internal consensus, validate the model with literature on the topic, mediation and structural equation modelling for more robust quantitative analysis)

Adapting GMB workshops to an online setting has its limitations. Aiming for small groups, for ease of facilitation and communication, can limit the diversity of perspectives about the problem [20]. GMB activities can be cognitively demanding and further exacerbated by screen fatigue [13, 20]. In our case, breaking down the activities into short workshops (i.e. 30 to 90 min) on different days enhanced participants’ engagement. Having GMB workshops fully online risks limiting participation of people with limited technological knowledge who might be discouraged to take part, it excludes people with no or poor internet access, and people living in space-limited accommodation might not feel comfortable participating due to restricted privacy whilst taking part in the workshop. It was difficult to reach minority groups and participants from different ethnicities or socio-economic backgrounds. This might have been due to having limited time for recruitment. Additionally, COVID-19 restrictions had just started, therefore the transition to online environments was still developing and potential participants might not have felt encouraged enough to participate in an online workshop. It would be especially valuable to explicitly design online GMB to include these groups which tend to be left out of online discussions and could further exacerbate social and health inequalities [28]. GMB workshops are designed to be delivered in person, and one could potentially miss the rapport built in a face-to-face setting. However, we believe that breaking down the activities into different workshops and having an initial individual introductory meeting allowed participants to become familiar with the concepts, the platforms and the problem before having the group activity. Due to COVID-19 we had very limited time with the practitioners and policymakers and they felt unsatisfied with how much they could contribute during the workshop. Having an initial introductory meeting (one-on-one) with them could save time during the group workshop and allow more time for discussing the map and policy ideas. Extending the group workshop by 30 min (1.5 h total) might also help.

Through a systems thinking approach and using an adapted GMB methodology, individuals were able to have a better understanding of the complex system that influences dietary behaviour in the light of food industry influence. By using systems thinking tools, adolescents confirmed their understanding of this complex system at the level of recognising that all the factors they mentioned were interconnected, they recognised their interdependencies and highlighted important reinforcing feedback loops when analysing system outcomes (i.e. a food system which gives preference to unhealthy vs healthy food choices), and suggested interventions to balance the system in favour of healthy food choices [25].

In our experience, designing and delivering GMB online is feasible, engaging, cost-saving and an enjoyable experience. Participants gave positive feedback in terms of engagement, enjoyment and it allowed them to recognise and accept different points of view about the same problem.

## Conclusions

Online GMB workshops are achievable and an enjoyable experience. Participants became familiarised with system thinking and system dynamics concepts, they developed a shared understanding of a complex issue and they were able to portray it in a system map that depicted the most important factors in a causal structure and how they interact at various levels. In Table 3 we share the main recommendations of the most important things to take into consideration when running an online GMB workshop.

Standardising online GMB approaches would be valuable to compare effectiveness and quality between online and in-person workshops [14, 29, 30]. Evaluations of online versus in-person GMB workshops will enrich our knowledge of the effect online system mapping workshops has on stakeholders, the trade-off between effectiveness, quality and efficiency, and how this might be enhanced to identify leverage points and achieve systemic changes in complex issues.

## Data Availability

The datasets supporting the conclusions of this article are included within the article.

## Abbreviations

CLD: Causal Loop Diagram
GMB: Group Model Building
SD: System dynamics
